# New species and new records of the genus *Scrobipalpa* Janse (Lepidoptera, Gelechiidae) from China

**DOI:** 10.3897/zookeys.840.30434

**Published:** 2019-04-17

**Authors:** Houhun Li, Oleksiy Bidzilya

**Affiliations:** 1 College of Life Sciences, Nankai University, Tianjin 300071, China Nankai University Tianjin China; 2 Institute for Evolutionary Ecology of the National Academy of Sciences of Ukraine, 37 Academician Lebedev str., 03143, Kiev, Ukraine Institute for Evolutionary Ecology of the National Academy of Sciences of Ukraine Kiev Ukraine

**Keywords:** Gnorimoschemini, new combination, new synonymy, Palaearctic Region, taxonomy

## Abstract

An annotated list of 71 species of the genus *Scrobipalpa* in China is given. Nine species of the genus *Scrobipalpa* Janse, 1951 are described as new: *S.triangulella***sp. n.** (Gansu, Ningxia, Shaanxi), *S.punctulata***sp. n.** (Henan, Shanxi), *S.septentrionalis***sp. n.** (Heilongjiang, Ningxia), *S.zhongweina***sp. n.** (Ningxia), *S.tripunctella***sp. n.** (Hebei, Ningxia, Shanxi), *S.ningxica***sp. n.** (Ningxia), *S.psammophila***sp. n.** (Ningxia), *S.zhengi***sp. n.** (Inner Mongolia, Ningxia), and *S.liui***sp. n.** (Shanxi). *Scrobipalpagorodkovi* Bidzilya, 2012 is synonymised with *S.subnitens* Povolný, 1967. The female of *S.flavinerva* Bidzilya & Li, 2010 is described for the first time. Two new combinations are proposed: *Scrobipalpaperfecta* (Povolný, 1996), **comb. n.**, *Scrobipalpasuaveolens* (Povolný, 1996), **comb. n.** Twelve species of *Scrobipalpa* are recorded from China for the first time. *Scrobipalpacryptica* Povolný, 1996, *S.proclivella* (Fuchs, 1886) and *S.smithi* Povolný & Bradley, 1964 are removed from the list of *Scrobipalpa* of China due to misidentification.

## Introduction

The genus *Scrobipalpa* Janse, 1951 is considered as the genus with most species in the tribe Gnorimoschemini, and one of the most diverse genera in the family Gelechiidae. Approximately 250 species are known in the Palaearctic Region (Povolný 2002; Bidzilya and Li 2010), 104 of which are found in Europe (Huemer and Karsholt 2010). Ten species, including four introduced from Europe, have been recorded from North America (Lee et al. 2009). The status of of ten Nearctic species remains unclear, some of these are still undescribed, others show close affinity to the Palaearctic species and need further study (Nazari and Landry 2012). More than 30 species are known in the Afrotropical Region (Bidzilya, unpublished), five species in Australia (Povolný 1977), and two in the Oriental Region.

The larvae of most species feed on Asteraceae and Amaranthaceae (Povolný 1990). The latter is a dominant plant family for *Scrobipalpa* species in the deserts of Uzbekistan and probably in other arid areas of the Palaearctic Region (Falkovitsh and Bidzilya 2006). Some species are known as pests on Solanaceae (Povolný 1991).

This is a further contribution towards the inventory of the Gnorimoschemini of China. The genera *Ephysteris* Meyrick, 1908, *Kiwaia* Philpott, 1930, *Gnorimoschema* Busck, 1900 and a part of *Scrobipalpa* Janse, 1951 were reviewed in previous papers (Li and Bidzilya 2008; Bidzilya and Li 2010, 2016; Li and Bidzilya 2017). Since the first list of the Chinese *Scrobipalpa*-species has been published (Bidzilya and Li 2010), the Insect Collection of Nankai University was supplemented with additional material from various provinces of China. As a result of study of this material, 22 species are added to the Chinese fauna, nine of them are described as new. The unknown female of *S.flavinerva* is described here.

The current list of Chinese *Scrobipalpa* comprises 71 species. Approximately ten other species are represented by single or worn specimens and remain under further study.

## Material and methods

The present study is based on material deposited in the Insect Collection, College of Life Sciences, Nankai University (NKU), Tianjin, China.

Adults were examined with an Olympus SZX16 stereo microscope. Male and female genitalia were prepared using the unrolling technique as described by Pitkin (1986) and Huemer (1988) or follow the methods introduced by Li (2002). Images of adults and genitalia were taken with a Leica M205A stereo microscope and Leica DM750 microscope, coupled with the Leica Application Suite 4.2 software, respectively.

Biological data were mainly extracted from bibliographic sources. The distribution of species was established primarily from the material examined and supplemented by data in the literature. The “Material” is arranged in geographical order from northwest to southeast and countries referred to by their current names. The type material of the new described taxa is deposited in NKU. The descriptive terminology of the genitalia structures generally follows Huemer and Karsholt (2010).

## Results

### Descriptions of species

#### Scrobipalpa triangulella sp. n.

Taxon classificationAnimaliaLepidopteraGelechiidae

http://zoobank.org/336EEE75-7B31-4788-BFF6-9A8DD32225A1

Figs 1 , 15 , 25

##### Type material.

**CHINA: Holotype** ♀, Botanical Garden, Mt. Liupan, Ningxia Hui Autonomous Region, 1900 m, 30.vi.2007, coll. Xinpu Wang (gen. slide no. L14034) (NKU). Paratypes: 2 ♂, 1 ♀, same data as for holotype (gen. slide nos. L07026♂, L14009♀); 1 ♀, Mt. Liupan, Ningxia, 1700 m, 1.vii.2008, coll. Shulian Hao and Zhiwei Zhang; 1 ♀, Mt. Liupan, Ningxia, 2050 m, 7.vii.2008, coll. Shulian Hao and Zhiwei Zhang; 2 ♂, Mt. Xinglong, Yuzhong County, Gansu Province, 2120, 2130 m, 2, 30.vii.1993, coll. Houhun Li (gen. slide nos. L14031, L13041); 1 ♀, Huoditang, Ningshan County, Shaanxi Province, 1620 m, 4.vii.1990, coll. Jinfu Li (gen. slide no. L06062).

##### Diagnosis.

The new species is well defined externally by the presence of a distinct black triangular subcostal spot. *S.caryocoloides* Povolný, 1977 has a more uniform brown forewing, a smaller and less distinct triangular costal spot and brown rather than light grey ground colour of the forewing. The male genitalia resemble those of *S.peteri* Bidzilya, 2009, but the uncus is broader, the valva is longer and the sacculus is shorter and narrower. The female genitalia resemble those of *S.lutea* Povolný, 1977, *S.pseudolutea* Piskunov, 1990 and *S.candicans* (Povolný, 1996) (see Povolný 2002, Pl. 76, figs 799, 800, 803), but can be recognized by the presence of teeth on the basal plate of the signum.

##### Description.

**Figures 1–8.**
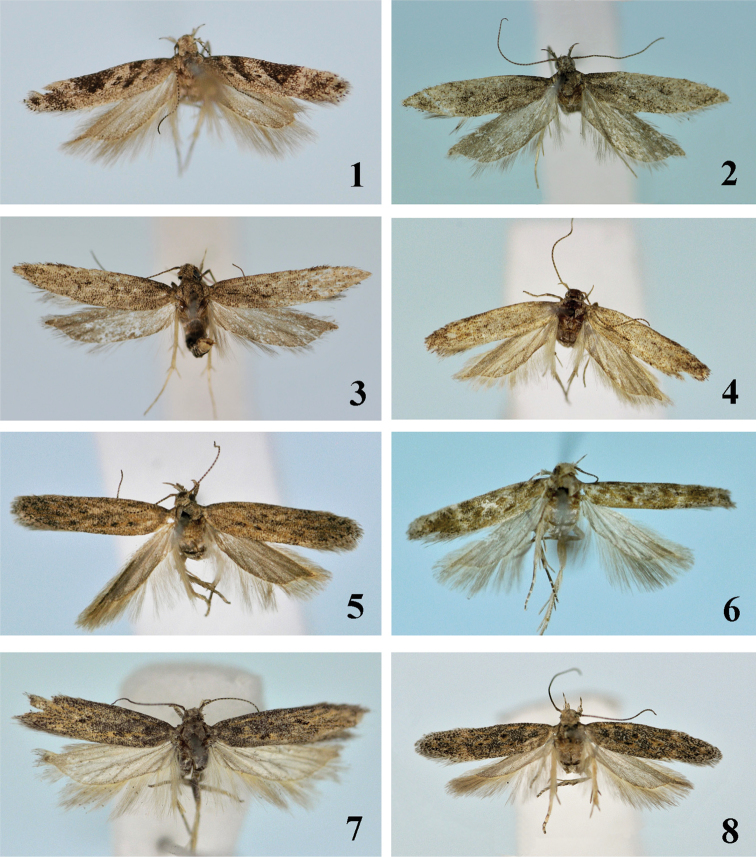
Adults of *Scrobipalpa* species. **1***S.triangulella* sp. n., HT, female (gen. slide no. L14034) **2***S.punctulata* sp. n., HT, male (gen. slide no. L13030) **3***S.punctulata* sp. n., PT, male **4***S.septentrionalis* sp. n., HT, male (gen. slide no. L07058) **5***S.septentrionalis* sp. n., PT, female (gen. slide no. L07030) **6***S.zhongweina* sp. n., PT, female (gen. slide no. 46/14, O Bidzilya) **7***S.tripunctella* sp. n., PT, male (gen. slide no. LLJ15208) **8***S.tripunctella* sp. n., PT, female (gen. slide no. L13061).

**Adult** (Fig. 1). Wingspan 12.0–14.0 mm. Head, thorax and tegulae light grey to black, frons nearly white, labial palpus up-curved, covered with white tipped black scales, segment 2 with brush of modified scales underside, inner and upper surface white, segment 3 ca. 1.5 times narrower and slightly shorter than segment 2, acute, antennal scape almost black, other antennal segments black with white basal belts; forewing light grey, black narrow oblique fascia form base to middle width, second black fascia from 1/3 of costal margin to 2/3 width, black triangular spot at 1/2–3/4 length of costal margin and to half width, apex mottled with black, black spot in fold, paired black point in cell, subapical facia light grey, cilia grey, black-tipped; hindwing grey.

**Male genitalia** (Fig. 15). Uncus twice as long as broad, rounded posteriorly; gnathos short, weakly curved; tegumen broad, anteromedial emargination deep, broadly rounded; valva narrow, of equal width, weakly curved, pointed apically, extending to the top of uncus; sacculus ca. 1/5 length of valva and slightly narrower than valva at base, with pointed inwardly curved tip, fused with vincular processes in all length except for distal 1/4–1/5, gap to vincular process small; vincular process broader and as long as sacculus, apex rounded with outwardly curved tip; posterior margin of vinculum with deep v-shaped medial emargination; saccus broad at base, then parallel-sided, apex knob-shaped, extending far beyond top of pedunculus; phallus of moderate width, straight, apical arm narrow, sinuous, caecum weakly inflated, slightly shorter than half length of phallus.

**Variation.** Uncus and saccus vary in width.

**Female genitalia** (Fig. 25). Papilla analis sub-ovate, sparsely covered with hairs; apophyses posteriores five times longer than segment VIII; sternite VIII weakly sclerotized, subgenital plates 1/3 width of segment VIII, parallel-sided, with a few folds along medial margin; ventromedial depression smooth, subrectangular posteriorly, trapezoid anteriorly, divided by triangular anteromedial emargination into paired lobes that extending slightly beyond anterior margin of sternite VIII; apophyses anteriores longer than segment VIII, straight; ductus bursae broad, of moderate width, colliculum narrow, ring-shaped; corpus bursae elongated, subovate, slightly shorter than ductus bursae; base of signum large with one big and a few very small teeth, distal hook narrow, weakly curved.

##### Distribution.

China (Gansu, Ningxia, Shaanxi).

##### Biology.

Host plant unknown. Adults were collected from late June to late July at altitudes from 1600 to 2200 m.

##### Etymology.

The species is named after the characteristic triangular costal spot on the forewing.

##### Remarks.

This species was erroneously associated with the female of *S.caryocoloides* (Bidzilya and Li 2010: 12, fig. 31). We found two females from the same locality that match well externally with the male (gen. slide L07026) figured in above cited paper and differ from the female of *S.caryocoloides* (see Povolný 2002: pl. 76, fig. 798; Park and Ponomarenko 2006: fig. 46; Bidzilya and Li 2010: 21, fig. 50). Therefore, neither male nor female in the present series are conspecific with *S.caryocoloides* and actually represent both sexes of a new species that is described here.

#### Scrobipalpa punctulata sp. n.

Taxon classificationAnimaliaLepidopteraGelechiidae

http://zoobank.org/A25FA39C-58E9-47E4-9384-11EC2ABA770B

Figs 2 , 3 , 16 , 17

##### Type material.

**CHINA: Holotype** ♂, Mt. Luya, Ningwu County, Shanxi Province, 1450 m, 24.vii.2011, coll. Shulian and Jiayu Liu (gen. slide no. L13030) (NKU). **Paratypes**: 6 ♂, 1 ♀ (abdomen missing), same data as for holotype, 19, 20, 24.vii.2011 (gen. slide no. 71/14, O Bidzilya); 1 ♂, Baotainman, Neixiang County, Henan Province, 1200 m, 23.v.2006, coll. Xu Zhang and Jinmei Lv (gen. slide no. L06110).

##### Diagnosis.

*Scrobipalpapunctulata* sp. n. externally does not show any diagnostic characters to separate it from congeneric species. The male genitalia are close to those of *S.triangulella* sp. n., *S.reiprichi* Povolný, 1984 and *S.corleyi* Huemer & Karsholt, 2010, but the vincular process is narrower, the gap to the sacculus is larger, the valva is shorter and the saccus is narrower; *S.reiprichi* has the uncus narrower, the sacculus and saccus broader; *S.corleyi* differs in a broader uncus and broader vincular process.

##### Description.

**Adult** (Figs 2, 3) Wingspan 12.0–15.0 mm. Head, thorax and tegulae grey, frons light grey, labial palpus grey mottled with black, inner surface of segment 2 off-white, scape grey, other antennal segments grey with narrow light grey basal rings; forewing plain greyish-brown, basal half of subcostal vein and fold yellowish-white, black spots in cell indistinct, cilia grey black tipped; hindwing and cilia grey.

**Male genitalia** (Figs 16, 17) Uncus elongated, posterior margin weakly emarginated; gnathos short, slender, weakly curved; tegumen broad, anteromedial emargination deep, broadly rounded; valva moderately narrow, nearly straight, weakly constricted in middle, apex rounded, extending to the top of uncus or slightly shorter; sacculus ca. 1/4 length of valva, outer margin rounded with pointed inwardly curved tip, gap to vincular process narrow; vincular processes slightly shorter than sacculus, distal portion narrow, apex with outwardly curved tip; posterior margin of vinculum with deep v-shaped medial emargination; saccus narrow, tapered, slightly extending beyond top of pedunculus; phallus of moderate width, straight, apical arm narrow, sinuous, caecum weakly inflated, 1/3 length of phallus.

**Variation.** Sacculus and vincular process vary slightly in width.

**Female.** Unknown.

##### Distribution.

China (Henan, Shanxi).

##### Biology.

Host plant unknown. Adults were collected in late May and July at altitudes of 1200–1450 m.

##### Etymology.

The species name is derived from the Latin adjective *punctulatus* (having small spots), referring to the spots on the forewing.

#### Scrobipalpa septentrionalis sp. n.

Taxon classificationAnimaliaLepidopteraGelechiidae

http://zoobank.org/F928984C-AE2E-4B61-B08C-504FEFFBA92D

Figs 4 , 5 , 18 , 26

##### Type material.

**CHINA: Holotype** ♂, Yuanyichang, Zhongning County, Ningxia Hui Autonomous Region, 17.vii.1993, 1170 m, coll. Houhun Li (gen. slide no. L07058) (NKU). Paratypes: 3 ♀, same data as for holotype (gen. slide nos. L07030, L13055, SYW05246); 2 ♀, Xinpu, Zhongning County, Ningxia, 26.vii.1993, 1170 m, coll. Houhun Li (gen. slide no. L93083); 3 ♀, Harbin, Heilongjiang, 150 m, 23.vii.1997, coll. Houhun Li (gen. slide nos. L14054, LLJ15219, 115/15, O Bidzilya).

##### Diagnosis.

This new species resembles *S.ningxica* sp. n. externally, but is larger, paler and with less well expressed dark irroration. The male genitalia resemble those of *S.alterna* Falkovitsh & Bidzilya, 2006 and *S.lutea* (see Huemer and Karsholt 2010, figs 65, 66), but the uncus is shorter, the valva is longer, the vincular process is strongly curved and the saccus is narrower. The female genitalia are similar to those of *S.pauperella* (Heinemann, 1870), *S.spumata* (Povolný, 2001) and *S.pseudolutea*, but differ in the absence of the foamy sculpture at base of the apophyses anteriores and the longer, weakly curved distal hook of the signum.

##### Description.

**Adult** (Figs 4, 5). Wingspan 11.0–13.5 mm. Head, thorax and tegulae covered with light grey brown tipped scales, frons white, labial palpus grey, segment 2 mottled with brown on outer surface and on underside, upperside white, segment 3 with light brown basal and medial belts, scape light brown, other antennal segments brown with whitish basal rings; forewing creamy grey, mixed with light brown particularly near apex, subcostal vein and fold mottled with yellow, three indistinct brown dots in fold, black spots in cell indistinct, cilia grey brown tipped; hindwing and cilia grey.

**Variation.** The ground colour of forewing varies from cream to light brown.

**Male genitalia** (Fig. 18). Uncus twice as long as broad, rounded in apical 1/3; gnathos short, weakly curved; tegumen prolonged with deep and broad anteromedial emargination; valva narrow, of equal width, weakly curved, apex rounded, extending over the top of uncus; sacculus 1.5 times broader and ca. 4 times shorter than valva, inner margin straight, outer margin weakly curved, apex rounded, tip pointed, curved inwards, gap to the vincular process moderately large; vincular process large, slightly broader and as long as sacculus, inner margin strongly curved, apex rounded with pointed tip; posterior margin of vinculum with deep and broad v-shaped medial emargination; saccus narrow, tapered towards rounded apex, extending far beyond apex of pedunculus; phallus straight, apical arm narrow, caecum strongly inflated, 1/3 length of phallus.

**Female genitalia** (Fig. 26). Papilla analis subtriangular, sparsely covered with short hairs; apophyses posteriores ca. 4.5 times longer than segment VIII; sternite VIII longer than broad, subtrapezoidal, subgenital plates 1/3 width of sternite VIII, smooth, broadened anteriorly, inner margin with distinct fold; ventromedial depression subrectangular, divided by short triangular anteromedial incision into broad lobes, which extends beyond anterior margin of sternite VIII; apophyses anteriores shorter or as long as segment VIII; ductus bursae narrow in posterior portion, then evenly broadened towards pyriform corpus bursae, colliculum narrow, belt-shaped; signum situated in middle of corpus bursae, basal plate small, distal hook long and narrow, curved in distal one third, with a few teeth near the base.

**Variation.** Number of teeth at base of the signum varies from 3 to 5, and the apophyses anteriores vary in length.

##### Distribution.

China (Heilongjiang, Ningxia).

##### Biology.

Host plant unknown. Adults were collected in July at an altitude of 1100–1200 m.

##### Etymology.

The species name is the Latin adjective *septentrionalis* (northern), referring to the distribution of the species in the northern part of China.

#### Scrobipalpa zhongweina sp. n.

Taxon classificationAnimaliaLepidopteraGelechiidae

http://zoobank.org/FD6B2080-367B-49DA-8019-1D7AECEA0773

Figs 6 , 19 , 27

##### Type material.

**CHINA: Holotype** ♂, Shapotou, Zhongwei County, Ningxia Hui Autonomous Region, 1200 m, 10.viii.2000, coll. Houhun Li and Shuxia Wang (gen. slide no. L06088) (NKU). **Paratype**: 1 ♀, same data as holotype (gen. slide no. 46/14, O Bidzilya).

##### Diagnosis.

The new species resembles externally the light-brown specimens of the rather variable *S.salinella* (Zeller, 1847) (see Huemer and Karsholt 2010, fig. 107), but the black irroration under the costal margin and near the apex is less distinct and the reddish spots are smaller. The male genitalia look similar to those of *S.grisea* Povolný, 1969 (see Huemer and Karsholt 2010, fig. 34), but the saccus and the vincular processes are narrower whereas the sacculus is broader in *S.grisea*. The female genitalia are similar to those of *S.thymelaeae* (Amsel, 1939) (see Huemer and Karsholt 2010, fig. 111), but the outer margin of the ventromedial depression is weakly edged, the apophyses anteriores are thinner and the ductus bursae is narrower in the distal portion.

##### Description.

**Adult** (Fig. 6). Wingspan 11.0 mm. Head, thorax and tegulae light brown, frons nearly white, labial palpus light brown with diffuse inner and subapical white belts, inner and upper surface whitish, underside of segment 2 with brush of modified scales, segment 3 nearly as long as segment 2, two times narrower, pointed; antennae brown ringed with grey; forewing covered with light grey brown-tipped scales and densely irrorate with reddish along dorsum, on basal half of subcostal vein and in subapical 1/4, costal margin and termen mottled with black, reddish streak and prolonged reddish spot in fold, three reddish-brown spots in cell, white subapical fascia at 3/4, cilia grey brown-tipped, hindwing grey.

**Male genitalia** (Fig. 19). Uncus twice as long as broad, rounded in apical 1/3; gnathos short, weakly curved; tegumen prolonged with deep and broad anteromedial emargination; valva curved in middle, apex rounded, extending to the top of uncus; sacculus weakly curved inwards, ca. 5 times shorter than valva, broad at base, then narrowing, tip curved inwards, gap to the vincular process rounded; vincular process short, beak-shaped; posterior margin of vinculum with deep v-shaped medial emargination; saccus narrow, pointed, extending far beyond the apex of pedunculus; phallus straight, apical arm narrow, caecum strongly inflated, approx. as long as phallus.

**Female genitalia** (Fig. 27). Papilla analis subovate, sparsely covered with short hairs; apophyses posteriores ca. 4.5 times longer than segment VIII; sternite VIII approx. as broad as long, weakly narrowing apically, anterior margin straight, subgenital plates 1/3 width of sternite VIII, parallel-sided with foamy sculpture along medial margin from half-length to the base of apophyses anteriores; ventromedial depression trapezoidal, divided by anteromedial triangular incision to 3/4 length into densely covered with microtrichia subovate lobes, which extending to the anterior margin of sternite VIII; apophyses anteriores longer than segment VIII; ductus bursae narrow in posterior portion, then evenly broadened towards globular corpus bursae, colliculum narrow, ring-shaped; signum situated near the entrance of corpus bursae, stout, basal plate small, distal hook weakly curved.

##### Distribution.

China (Ningxia).

##### Biology.

Host plant unknown. Adults fly in July at an altitude of 1200 m.

##### Etymology.

The species name, a noun in apposition, is derived from the type locality: Zhongwei County, Ningxia Hui Autonomous Region.

#### Scrobipalpa tripunctella sp. n.

Taxon classificationAnimaliaLepidopteraGelechiidae

http://zoobank.org/61285548-7CC4-4576-BEAD-F87955F618D3

**Figures 9–14.**
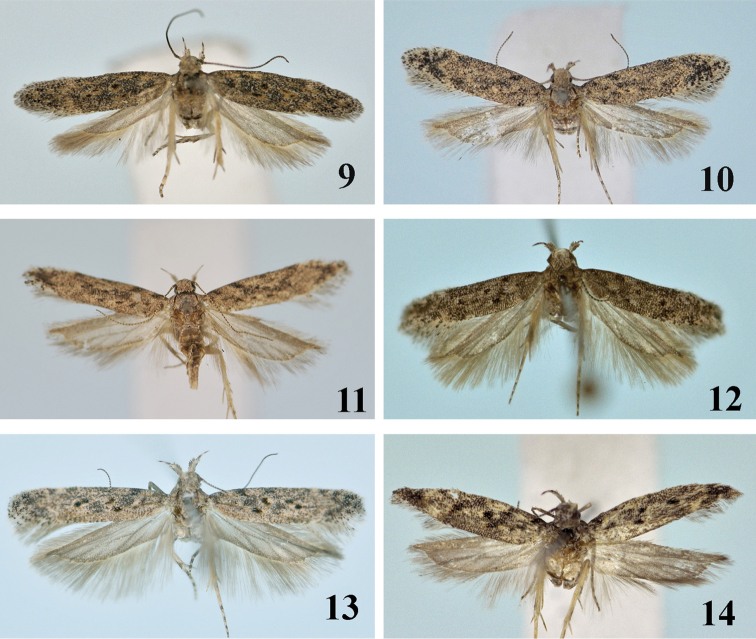
Adults of *Scrobipalpa* species. **9***S.tripunctella* sp. n., PT, female (gen. slide no. L14056) **10***S.ningxica* sp. n., PT, female (gen. slide no. L13092) **11***S.ningxica* sp. n., Paratype, female **12***S.psammophila* sp. n., HT, male (gen. slide no. L07045) **13***S.zhengi* sp. n., HT, male (gen. slide no. 125/08. O Bidzilya) **14***S.liui* sp. n., HT, male (L14058).

Figs 7–8 , 9 , 20 , 28

##### Type material.

**CHINA: Holotype** ♂, Huangjijuan, Yanchi County, Ningxia Hui Autonomous Region, 17.vi.2014, coll. Houhun Li, Wei Guan and Meiqing Yang (gen. slide no. L13074) (NKU). **Paratypes**: 1 ♂, same data as holotype (gen. slide no. L13079); 4 ♀, Qiuqianjia, Mt. Liupan, Ningxia, 1700 m, 2.vii.2008, coll. Shulian Hao and Zhiwei Zhang (gen. slide nos. 96/15, O Bidzilya; LLJ15213; LLJ15223); 1 ♀, Guamagou Forest Farm, Mt. Liupan, Ningxia, 29.vi.2008, 2100 m, coll. Shulian Hao and Zhiwei Zhang (gen. slide no. L13061); 1 ♂, Guanliju, Mt. Liupan, Ningxia, 1700 m, 20.vi.2008, coll. Shulian Hao and Zhiwei Zhang (gen. slide no. LLJ15208); 1 ♀, Fengtai Forest Farm, Mt. Liupan, Ningxia, 12.vii.2008, 2330 m, coll. Shulian Hao and Zhiwei Zhang (gen. slide no. 122/13, O Bidzilya); 1 ♀, Qipanshan, Weichang County, Hebei Province, 17.vii.2001, coll. Yanli Du and Shulian Hao (gen. slide no. SYW05256); 2 ♀, Xizhashui, Linchuan County, Jincheng, Shanxi Province, 13.vii.2010, 900 m, coll. Haiyan Bai and Linlin Yang (gen. slide no. L14056).

##### Diagnosis.

The new species resembles externally *S.ningxica* sp. n., but it is darker and has no white subapical fascia. The male genitalia are close to those of *S.heimi* Huemer & Karsholt, 2010, but the sacculus is longer, the vincular process is weakly curved and the sacculus is narrower. The female genitalia are most similar to those of *S.amseli* Povolný, 1966, but segment VIII is longer, its anterior margin is straight rather than emarginated, the lobes of the ventromedial depression are shorter and the signum is larger.

##### Description.

**Adult** (Figs 7–9). Wingspan 15.5–17.0 mm. Head, thorax and tegulae light brown, frons light grey, labial palpus light grey, outer surface mottled with brown, underside of segment 2 with groove; scape brown, other antennal segments brown, ringed with grey; forewing covered with light grey brown-tipped scales, distal 1/2–1/3 lighter, cream mixed with light brown, subcostal vein in basal 1/2 and fold yellowish-brown, three black spots in cell: two in middle and another paired in the corner, cilia grey black tipped, hindwing and cilia light grey.

**Variation.** The ground colour of forewing varies from light cream to light brown, and the black marking is rather poorly expressed in some specimens.

**Male genitalia** (Fig. 20). Uncus broader than long, rounded in apical 1/3; gnathos short, weakly curved; tegumen moderately prolonged with deep and broad anteromedial emargination; valva approx. of equal width, evenly curved, apex inflated, rounded, extending to the top of uncus; sacculus nearly twice as broad and 4–5 times shorter than valva, subrectangular, outer margin with rounded apex, tip pointed, gap to the vincular process large, rounded; vincular process shorter and narrower than sacculus, outwardly curved; posterior margin of vinculum with broad v-shaped medial emargination; saccus broad, of even width, apex truncated, extending slightly beyond the apex of pedunculus; phallus narrow, straight, apical arm narrow, strongly curved distally, caecum strongly inflated, ca. 1/3 length of phallus.

**Female genitalia** (Fig. 28). Papilla analis subovate, sparsely covered with short hairs; apophyses posteriores ca. 4.5 times longer than segment VIII; sternite VIII ca. as broad as long, anteromedial edge concave, anterior margin straight, subgenital plates broad in posterior half, strongly narrowing anteriolaterally with foamy sculpture along medial margin from half-length to the base of apophyses anteriores; ventromedial depression trapezoid, densely covered with microtrichia, folded laterally, anterior margin with triangular emargination, not extending to the anterior margin of sternite VIII; apophyses anteriores as long as segment VIII; ductus bursae moderately broad, colliculum narrow, ring-shaped, corpus bursae long, pyriform; basal plate of signum broad, rounded, distal hook broad at base, with three teeth, curved in middle, distal portion narrow, straight, pointed.

##### Distribution.

China (Hebei, Ningxia, Shanxi).

##### Biology.

Host plant unknown. Adults were collected from late June to early July at an altitude of 1700 m.

##### Etymology.

The species name, an adjective, is derived from the Latin prefix *tri*- (three), *punctus* (spot), and suffix -*ellus* referring to the forewing with three spots in the cell.

##### Remarks.

The conspecificity of males and females is proven by study of the specimens of both sexes that externally match each other and were collected in close localities in Mt Liupan: 20.vi (male) and 2.vii.2008 (female). *Scrobipalpapunctulata* differs in less distinct wing pattern and in the smaller size of adults.

#### Scrobipalpa ningxica sp. n.

Taxon classificationAnimaliaLepidopteraGelechiidae

http://zoobank.org/FDA43D78-A3A1-43F6-BD9B-7807FB3026BF

Figs 10 , 11 , 21 , 29

##### Type material.

**CHINA: Holotype** ♂, Erdaohu, Habahu, Yanchi County, Ningxia Hui Autonomous Region, 20.vii.2013, 1397 m (gen. slide no. L13097) (NKU). **Paratypes**: 10 ♂, 26 ♀, same data as holotype (gen. slide nos. L14042♂; L14008♀; L14019♀; L13092♀); 1 ♀, Habahu, Yanchi County, Ningxia, 24.vii.2013, 1461 m (gen. slide no. L14008).

##### Diagnosis.

The new species is defined externally by its nearly plainly coloured light grey to cream-coloured forewing densely mottled with black. The male genitalia resemble those of *S.indignella* (Staudinger, 1879) (see Huemer and Karsholt 2010: 392, fig. 68), but differ in the longer and narrower valva, the broader, U-shaped rather than V-shaped posteromedial emargination of the vinculum, the broader and apically inflated saccus as well as the broader and shorter phallus with more broadened apex. The female genitalia are most similar to those of *S.chrysanthemella* (O. Hofmann, 1867) (see Huemer and Karsholt 2010: 461, fig. 28), but differ in the presence of foamy sculpture at base of the apophyses anteriores, the strongly sclerotized and broad medially separated lobes of the ventromedial depression as well as more curved distal hook of the signum.

##### Description.

**Adult** (Figs 10, 11). Wingspan 11.0–12.5 mm. Head light brown to yellow, neck occasionally mottled with brown, labial palpus grey mixed with brown, inner surface of segment 2 light, off-white, underside with row of modified brown-tipped scales, segment 3 distinctly narrower and shorter than segment 2, acute, light grey, apex mixed with brown mainly in distal half, scape greyish brown, other antennal segments cream with brown rings; thorax and tegulae grey, brown tipped; forewing covered with cream to light grey, black-tipped scales, costal margin and termen slightly darker, white subapical fascia poorly expressed, fold yellowish with two black spots inside, three indistinct black dots in cell, diffuse black spot at base and on 1/4 of costal margin, dorsal margin with a few black scales at base; cilia grey black tipped; hindwing grey.

**Variation.** Forewing vary from light grey to dark grey.

**Male genitalia** (Fig. 21). Uncus twice as long as broad, weakly narrowing apically, posterior margin rounded; gnathos short, thin, weakly curved; basal half of tegumen broad, triangular, distal half parallel-sided, weakly narrower than uncus, anteromedial emargination deeply rounded; valva thin, of even width, evenly curved, apex inflated, rounded, extending to the top of uncus; sacculus four times shorter and twice broader than valva, inner margin straight with pointed tip curved inwards, outer margin slightly curved, apically rounded, gap to the vincular process moderately broad; vincular processes twice narrower and slightly shorter than sacculus, with outwardly curved and strongly pointed tip, medial emargination of posterior margin of vinculum deep, broadly rounded; saccus stout, broad, parallel-sided except for basal 1/3, apex broadened, projecting far beyond apex of pedunculus; phallus straight, apical arm narrow, caecum weakly inflated, ca. 1/3 length of phallus.

**Female genitalia** (Fig. 29). Papilla analis elongated, covered with short hairs; apophyses posteriores ca. 4 times longer than apophyses anteriores; segment VIII as broader as long, weakly narrowing apically, anterior margin straight, strongly sclerotized, subgenital plates posteriorly ca. half as broad as sternite VIII, strongly narrowing anteriorly, with foamed sculpture at base of apophyses anteriores, ventromedial depression smooth, strongly edged anteriorly, separated by medial triangular emargination into lobes reaching to the anterior margin of sternite VIII; apophyses anteriores straight, ca. as long as sternite VIII; posterior part of ductus bursae narrow with broad strongly laterally sclerotized colliculum, ductus bursae gradually widened towards pyriform corpus bursae; signum near entrance of corpus bursae, basal plate of signum large, distal hook narrow, curved under the right angle in middle.

##### Distribution.

China (Ningxia).

##### Biology.

Host plant unknown. Adults were collected in the last third of July at an altitude of 1400 m.

##### Etymology.

The specific name, a noun in apposition, refers to the type locality of the new species, Ningxia Hui Autonomous Region of China.

#### Scrobipalpa psammophila sp. n.

Taxon classificationAnimaliaLepidopteraGelechiidae

http://zoobank.org/678C542E-B41B-44F1-8C2B-60A2C98565ED

Figs 12 , 22

##### Type material.

**CHINA: Holotype** ♂, Botanical Garden, Mt. Liupan, Ningxia Hui Autonomous Region, 1900 m, 30.vi.2007 (gen. slide no. L07045) (NKU). **Paratype**: 1 ♂, same data as holotype (gen. slide no. 88/14, O Bidzilya).

##### Diagnosis.

The new species can hardly be recognized by external characters only. It is similar to *S.concerna* Povolný, 1969, *S.frugifera* Povolný, 1969, *S.tenebrata* Povolný, 2001 and other greyish brown species with black markings. The male genitalia are characterized by the deep and broad posteromedial emargination of the vinculum in combination with the strongly curved vincular process. *Scrobipalpakaszabi* Povolný, 1969 is somewhat similar in this respect (see Povolný 2002, Pl. 69, fig. 683), but its vincular process is much broader.

##### Description.

**Adult** (Fig. 12). Wingspan 12.0–12.2 mm. Head greyish-brown, frons white, labial palpus grey mottled with brown, inner and upper surface of segment 2 white, underside with brush of modified scales, segment 3 slightly shorter and ca. 1/2 as broad as segment 2, pointed, with indistinct whitish ring in middle, apex with few white scales, scape brown mixed with grey, other antennal segments brown, ringed with white at base; forewing, thorax and tegulae covered with grey brown-tipped scales, a few black scales on base, black dot on 1/3 length of costa, diffuse black spots in mid width and on dorsum near base, three black spots in cell, subcostal vein and fold mottled with light brown scales, diffuse costal and tornal white spots at 3/4, subapical 1/5 and termen mixed with white scales, cilia grey black tipped, hindwing grey.

**Male genitalia** (Fig. 22). Uncus clearly separated from tegumen, prolonged, tapered apically, slightly constricted in middle, apex rounded; gnathos short, narrow, apex weakly curved, pointed; tegumen prolonged, anterior emargination deep, rounded; valva slender, base and apex weakly dilated, reaching the top of uncus; sacculus ca. 1/3 length of valva, nearly of even width, inner margin slightly concave before pointed and inwardly curved apex, gap to vincular process long and narrow; vincular process as long and distinctly broader than sacculus, inner margin strongly curved, apex rounded with pointed tip; posterior margin of vinculum with very deep and broad subovate medial emargination; saccus narrow, tapered towards rounded apex, extending far beyond apex of pedunculus; phallus straight, broad, apical arm sinuous, narrow, caecum strongly inflated, slightly shorter than 1/2 length of phallus.

**Variation.** The length of uncus and width of vincular process vary slightly.

**Female.** Unknown.

##### Distribution.

China (Ningxia).

##### Biology.

Host plant unknown. Adults were collected in late June at an altitude of 1900 m in arid and semi-arid desert environment.

##### Etymology.

The species name, an adjective is derived from the Greek *psammos*, meaning sand, and the Greek verb *philein*, to love, referring to the habitats of the species being restricted to desert sandy environment.

**Figures 15–18.**
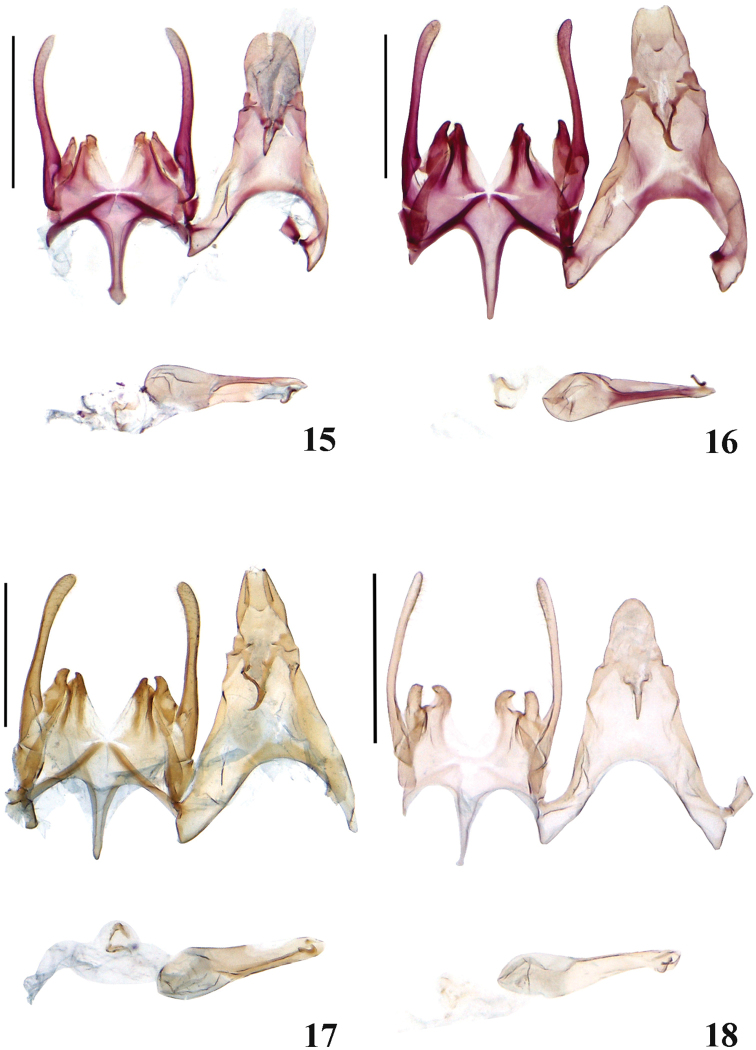
Male genitalia of *Scrobipalpa* species. **15***S.triangulella* sp. n., PT (gen. slide no. L13041) **16***S.punctulata* sp. n., HT (gen. slide no. L13030) **17***S.punctulata* sp. n., PT (gen. slide no. 71/14, O Bidzilya) **18***S.septentrionalis* sp. n., HT (gen. slide no. L07058). Scale bars: 0.5 mm (**15, 16, 18**); 0.2 mm (**17**).

#### Scrobipalpa flavinerva

Taxon classificationAnimaliaLepidopteraGelechiidae

Bidzilya & Li, 2010

Fig. 30

Scrobipalpa flavinerva Bidzilya & Li, 2010: 16, figs 15, 38.

##### Material examined.

3 ♂, 4 ♀, Erdaohu, Yanchi County, Ningxia Hui Autonomous Region, 1397 m, 19,20.vii.2013, coll. Houhun Li et al. (gen. slide nos. L13070♀; YMQ13167♂; LLJ15203♀).

**Female genitalia** (Fig. 30). Papilla analis elongate, ovate, sparsely covered with hairs; apophyses posteriores five times longer than segment VIII; sternite VIII twice as long as broad, weakly narrowing apically, postgenital plates smooth with stronger sclerotized zone along medial margin, rounded posteromedially, posteriorly ca. 1/3 width of sternite VIII, then gradually narrowing towards apophyses anteriores, ventromedial depression prolonged, trapezoidal, densely covered with microtrichia, with distinct lateral folds, divided anteromedially to 1/3 length into lobes which not extend beyond anterior margin of sternite VIII; apophyses anteriores as long as sternite VIII, straight; ductus bursae narrow to moderate width, with distinct transition to subovate corpus bursae, colliculum narrow, ring-shaped; signum stout, basal plate large, distal hook curved under the right angle, narrow, acute.

##### Biology.

Host-plant unknown. Adults were collected from the end of June to middle August at an altitude of 1400 m.

##### Distribution.

China (Ningxia, Inner Mongolia), Mongolia, Russia (Buryatia) (Bidzilya and Nupponen 2018).

##### Remarks.

This species was described from four males collected in Mongolia and Inner Mongolia in China. The female of this species is described for the first time based on specimens from Ningxia Hui Autonomous Region.

The female genitalia are very similar to those of *S.parki* (Povolný, 1993) (see Povolný 2002, Pl. 75, fig. 789), but can be recognized by the shorter lobes of the ventromedial depression not extending beyond the anterior margin of sternite VIII, as in the latter species. An additional difference is the broader basal plate of the signum.

**Figures 19–24.**
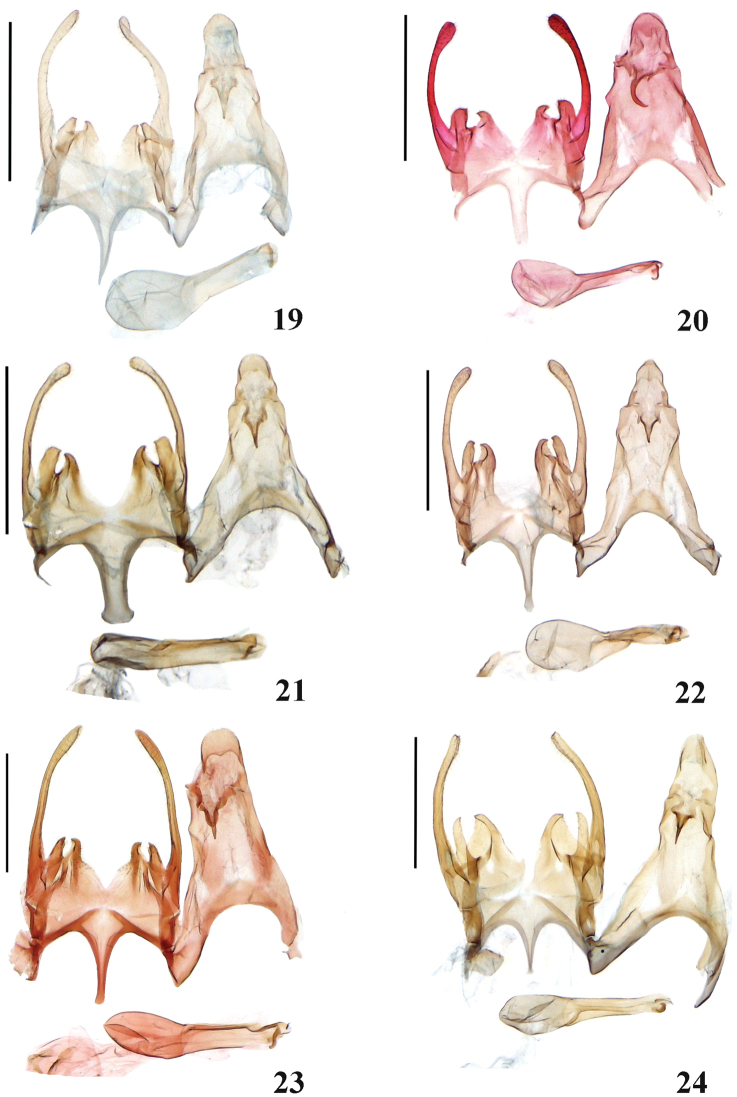
Male genitalia of *Scrobipalpa* species. **19***S.zhongweina* sp. n., HT (gen. slide no. L06088) **20***S.tripunctella* sp. n., PT (gen. slide no. LLJ15208) **21***S.ningxica* sp. n., HT (gen. slide no. L13097) **22***S.psammophila* sp. n., HT (gen. slide no. L07045) **23***S.zhengi* sp. n., HT (gen. slide no. 125/08. O Bidzilya) **24***S.liui* sp. n., HT (L14058). Scale bars: 0.5 mm.

#### Scrobipalpa zhengi sp. n.

Taxon classificationAnimaliaLepidopteraGelechiidae

http://zoobank.org/5A27CE1D-9D95-483D-856B-9DE4F27F6E35

Figs 13 , 23 , 31

Scrobipalpa cryptica Povolný, 1996, Bidzilya and Li 2010: 3. Misidentification.

##### Type material.

**CHINA: Holotype** ♂, Suyukou, Mt. Helan, Ningxia Hui Autonomous Region, 2000 m, 10.viii.2005, coll. Xinpu Wang et al. (gen. slide no. 125/08) (NKU). Paratypes: 1 ♀, same data as for holotype (gen. slide no. 34/14, O Bidzilya); 1 ♂, Xiazigou, Yaba, Mt. Helan, Alashan Left Banner, Inner Mongolia, 1793 m, 30.vii.2010, coll. Hongxia Liu and Zhiwei Zhang (gen. slide no. LJ17310).

##### Diagnosis.

The new species can hardly be separated externally from *S.cinerea* Povolný, 1996. The male genitalia remotely resemble those of *S.atriplicella* (Fischer von Röslerstamm, 1841), but can be distinguished by the shorter uncus, the tapered sacculus, the broader vincular process and the broader saccus. The female genitalia are similar to those of *S.sattleri* Lvovsky & Piskunov, 1989, but the subgenital plates are densely covered with microtrichia and the signum is smaller.

##### Description.

**Adult** (Fig. 13). Wingspan 14.5–15.0 mm. Head, thorax and tegulae covered with brown, black-tipped scales, frons white, labial palpus grey mottled with brown, segment 2 with diffuse black rings on outer surface, segment 3 with distinct broad black basal and medial rings, upper and inner surface of segment 2 dirty-white, scape brown, other antennal segments brown with light grey basal rings; forewing covered with grey black tipped scales, three diffuse black spots at base, black triangular spot on 1/5 of costal margin, paired black dot in fold on 1/4 length, black spot edged with ochreous on 1/4 length in middle, two others at 2/3 in middle width, diffuse strongly angulated light grey subapical fascia at 3/4 length, cilia greyish brown tipped; hindwing and cilia grey.

**Male genitalia** (Fig. 23). Uncus slightly longer than broad, subrectangular, posterolateral corners rounded; gnathos short, slender, weakly curved; tegumen narrow, anteromedial emargination extending to 1/3 length, broadly rounded; valva narrow, of even width, weakly curved, apex rounded, extending to the top of uncus; sacculus narrow, straight, ca. 1/5 length of valva, gradually tapered, apex pointed, inner margin with small hump before top; vincular process as long as sacculus, apex with outwardly curved tip; posterior margin of vinculum with deep v-shaped medial emargination; saccus broad at base, distal half narrow, of equal width, extending beyond the top of pedunculus; phallus straight, apical arm short, apex weakly pointed, caecum slightly broader and slightly longer than half length of phallus.

**Female genitalia** (Fig. 31). Papilla analis elongate, ovate, sparsely covered with hairs; apophyses posteriores five times longer than segment VIII; sternite VIII longer than broad, subrectangular, posterior margin weakly emarginated, postgenital plates ca. 1/3 width of sternite VIII, densely covered with microtrichia except for narrow area under posterior margin, gradually narrowing anteriorly, with gradual transition to apophyses anteriores, ventromedial depression prolonged, subrectangular, weakly narrowed in middle, densely covered with microtrichia, broadly divided anteromedially nearly to 1/2 length into edged lobes which not are extending beyond anterior margin of sternite VIII; apophyses anteriores as long as sternite VIII, straight, broad at base, gradually tapered; ductus bursae broad, of even width except for posterior portion that is narrower, with distinct transition to rounded corpus bursae; basal plate of signum small, distal hook short, weakly curved, narrow, acute.

##### Biology.

Host plant unknown. The adult was collected in early August at an altitude of 2000 m.

##### Distribution.

China (Inner Mongolia, Ningxia).

##### Etymology.

The species is named in honour of Professor Zhemin Zheng (Xi’an, China) for his outstanding contributions to systematic entomology.

**Figures 25–28.**
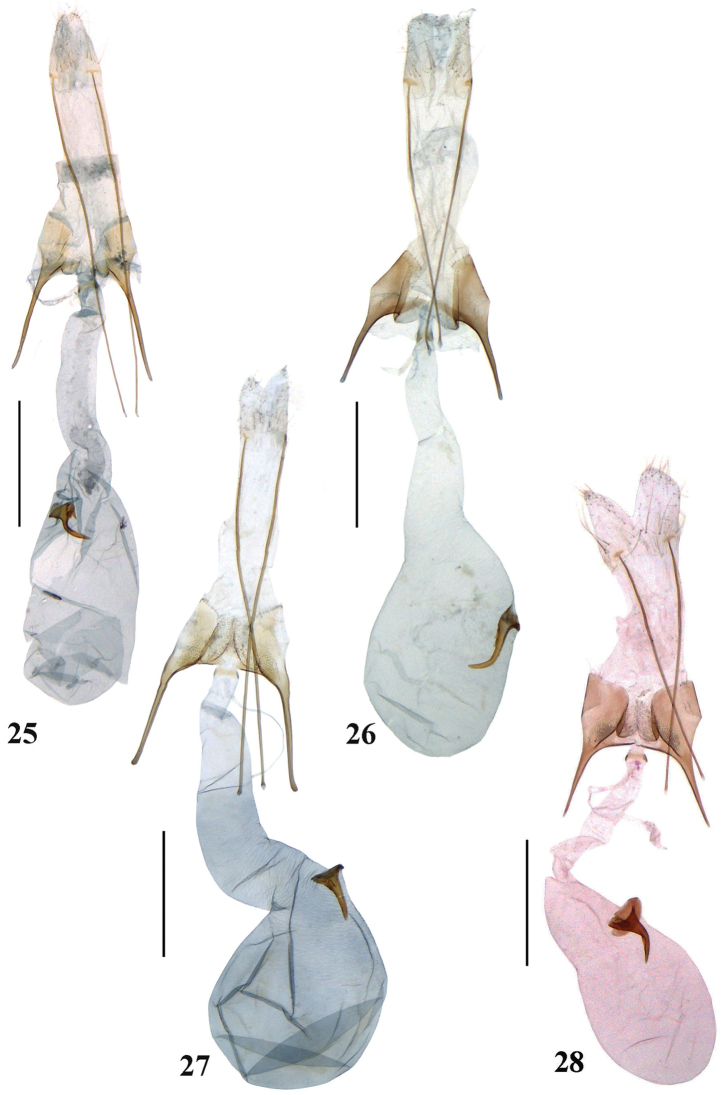
Female genitalia of *Scrobipalpa* species. **25***S.triangulella* sp. n., HT (gen. slide no. L14034) **26***S.septentrionalis* sp. n., PT (gen. slide no. L17030) **27***S.zhongweina* sp. n., PT (gen. slide no. 46/14, O Bidzilya) **28***S.tripunctella* sp. n., PT (gen. slide no. 96/15, O Bidzilya). Scale bars: 0.5 mm.

#### Scrobipalpa liui sp. n.

Taxon classificationAnimaliaLepidopteraGelechiidae

http://zoobank.org/C3F5B35E-DC65-4F9D-BE0D-76DD400D68C5

Figs 14 , 24

##### Type material.

**CHINA: Holotype** ♂, Manghe Macaques National Nature Reserve, Yangcheng County, Shanxi Province, 3.viii.2013, coll. Shulian Hao and Yanjun Fan (gen. slide no. L14058) (NKU).

##### Diagnosis.

The new species resembles externally *S.zhengi* and *S.cinerea*, but the black spots are more distinct. The male genitalia remotely resemble those of *S.spumata*, but the sacculus and the saccus are narrower and the vincular process is longer.

##### Description.

**Adult** (Fig. 14). Wingspan 12.0 mm. Head, thorax and tegulae grey mixed with brown, frons white, labial palpus grey mottled with brown, upper surface of segment 2 white, scape brown, other antennal segments brown with narrow light grey basal rings; forewing covered with grey brown-tipped scales, black spots at base and on 1/3 of costal margin, another black spot in mid width, fold mottled with yellow, three black diffuse spots surrounded by light brown scales in cell, indistinct subapical white fascia on 3/4, subapical area mottled with black and light brown, cilia greyish brown tipped; hindwing and cilia grey.

**Male genitalia** (Fig. 24). Uncus elongated, gradually narrowing in distal portion; gnathos short, slender, weakly curved; tegumen narrow, anteromedial emargination deep, broadly rounded; valva moderately narrow, weakly curved, apex rounded, extending to the top of uncus; sacculus narrow, 1/3 length of valva, straight, outer margin rounded, apex with pointed inwardly curved tip, gap to vincular process subtriangular; vincular process as long as sacculus, very broad, inner margin strongly sclerotized, gradually curved, apex with outwardly curved tip; posterior margin of vinculum with deep v-shaped medial emargination; saccus narrow, tapered, not extending beyond the top of pedunculus; phallus long, straight, apical arm short, strongly curved, apex pointed, caecum weakly inflated, 1/3 length of phallus.

**Female.** Unknown.

##### Biology.

Host plant unknown. The adult was collected in early August.

##### Distribution.

China (Shanxi).

##### Etymology.

This new species is named in honour of the late Professor Youqiao Liu (Beijing, China), who was a famous expert of Lepidoptera in China.

**Figures 29–31.**
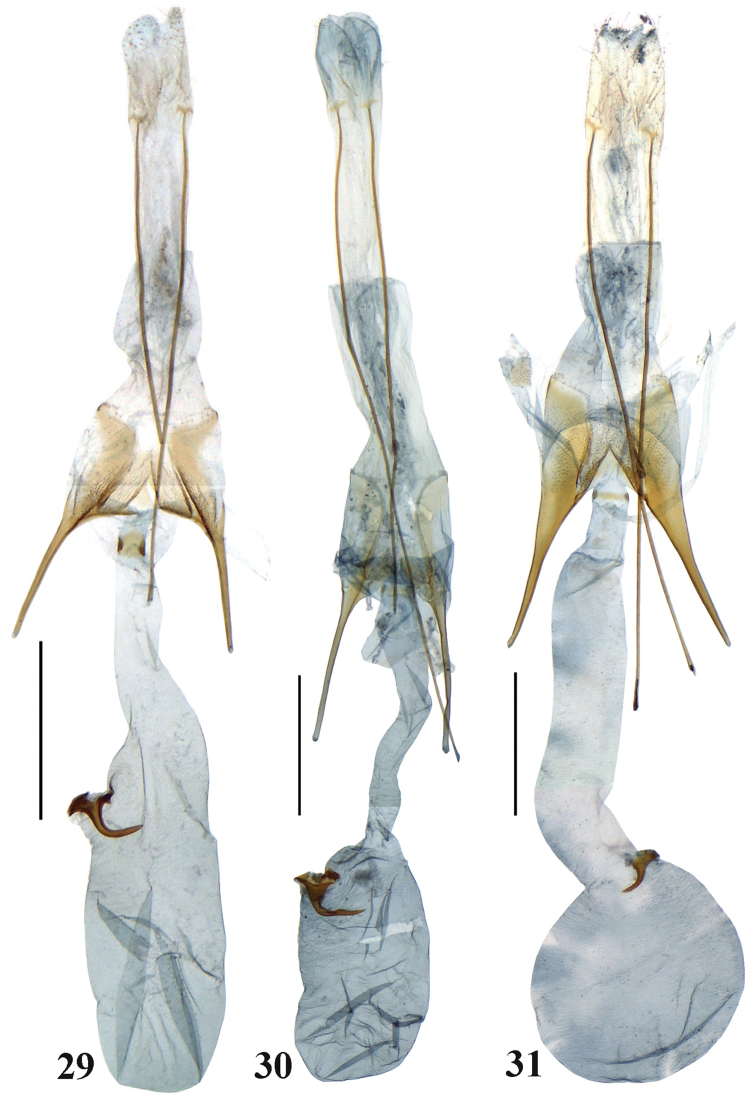
Female genitalia of *Scrobipalpa* species. **29***S.ningxica* sp. n., PT (gen. slide no. L13092) **30***S.flavinerva* (gen. slide no. L13070) **31***S.zhengi* sp. n., PT (gen. slide no. 34/14, O Bidzilya). Scale bars: 0.5 mm.

### An annotated list of the genus *Scrobipalpa* in China

**1. *Scrobipalpaaptatella* (Walker, 1864)**

**Distribution**. China (Xinjiang), east Australia, introduced into the Oriental and Afrotropical regions. Records from Europe are unconfirmed (Huemer and Karsholt 2010: 59).

**2. *Scrobipalpaacuminatella* (Sirkom, 1850)**

**Distribution**. China (Anhui), Europe, Russia (European part, Novosibirsk region, Kemerovo region, Irkutsk region), north Iran, Afghanistan, west Kazakhstan.

**3. *Scrobipalpaobsoletella* (Fischer von Röslerstamm, 1839)**

**Distribution**. China (Gansu, Inner Mongolia, Xinjiang), Palaearctic Region, South Africa, USA (introduced).

**4. *Scrobipalpaintricata* Povolný, 1969**

**Material examined**. 1 ♀, Kashgar, Xinjiang Uigur Autonomous Region, 17.ix.[19]82 (gen. slide no. 42/07, O Bidzilya).

**Distribution.** Mongolia, China (Xinjiang).

**Remarks.** This species is recorded from China for the first time.

**5. *Scrobipalpaatriplicella* (Fischer von Röslerstamm, 1839)**

**Distribution**. China (Henan, Heilongjiang, Inner Mongolia, Jilin, Qinghai, Shaanxi, Sichuan, Tibet, Xinjiang), Palaearctic Region, USA (introduced).

**6. *Scrobipalpavartianorum* Povolný, 1968**

**Distribution**. China (Xinjiang), Iran.

**7. *Scrobipalpaperfecta* (Povolný, 1996), comb. n.**

**Material examined.** 1 ♀, Qiongbola, Qapqal, Xinjiang Uigur Autonomous Region, 1871 m, 2.viii.2007, coll. Xinpu Wang et al. (gen. slide no. 51/14, O Bidzilya); 1 ♂, Qiongbola, Qapqal, Xinjiang, 3.viii.2007, 1480 m, coll. Xinpu Wang et al. (gen. slide no. L14025).

**Distribution.** China (Xinjiang), Kyrgyzstan.

**Remarks.** Both specimens differ superficially from *S.perfecta* figured by Povolný (2002, Pl. 5, fig. 1) in the more plainly coloured forewing without a light brown area along the dorsum. However, the specimens from China match the specimens from the type locality in respect to the genitalia.

**Remarks.** This species is recorded from China for the first time.

**8. *Scrobipalpasuaveolens* (Povolný, 1996), comb. n.**

**Material examined.** 2 ♂, Hemu, Burqin County, Xinjiang Uigur Autonomous Region, 23.vii.2007, 1114 m, coll. Xinpu Wang et al. (gen. slide nos. L13028; 122/15, O Bidzilya).

**Distribution.** China (Xinjiang), Kyrgyzstan.

**Discussion.** The male genitalia fit those of *S.suaveolens* well, except for the valva that extends over the top of the uncus and the uncus being slightly longer than that figured by Povolný (2002, Pl. 12, fig. 110). This may be the result of the different ways of genital preparation: the unrolling technique used by us and the traditional method used by Povolný. Externally the Chinese specimens differ in the absence of a black spot in the corner of the cell. However, the very characteristic narrow forewing with distinct pattern and the overall similar male genitalia leaves no doubt about the conspecificity of the Chinese specimens with *S.suaveolens*.

**Remarks.** This species is recorded from China for the first time.

**9. *Scrobipalpazhengi* sp. n.**

**Distribution.** China (Inner Mongolia, Ningxia).

**10. *Scrobipalpagrisea* Povolný, 1969**

**Distribution**. China (Inner Mongolia), Russia (Penza region, south Urals, Novosibirsk region, Tuva Republic, Zabaikalskiy krai), Mongolia, South Korea.

**11. *Scrobipalpaocculta* (Povolný, 2002)**

*Scrobipalpaproclivella* (Fuchs, 1886), Li 2002: 193. Misidentification.

*Scrobipalpasmithi* Povolný & Bradley, 1964; Bidzilya and Li 2010: 7. Misidentification.

**Material examined.** 2 ♂, 2 ♀, Tacheng, Xinjiang Uigur Autonomous Region, 17–30.vii.1990, coll. Jinfu Li (gen. slide no. L92065♂, L13034♀, 375/14♂, 379/14♀, O. Bidzilya); 1 ♂, Balian, Mohe Xiang, Gongliu County, Xinjiang, 5.viii.2007, 1206 m, coll. Xinpu Wang et al. (gen. slide no. L13052); 1 ♀, Weihugou, Jimsar County, Xinjiang, 10.viii.2007, 1734 m, coll. Xinpu Wang et al. (gen. slide no. 29/14, O. Bidzilya).

**Distribution.** China (Xinjiang), Russia (Volgograd region and Chelyabinsk region, Altai), Turkey.

**Remarks.** The species was mentioned from China as *S.proclivella* and as *S.smithi* respectively (Li 2002; Bidzilya and Li 2010). Here it is re-identified in accordance with the revision of the European Gnorimoschemini (Huemer and Karsholt 2010).

**12. *Scrobipalpakaszabi* Povolný, 1969**

**Material examined**. 1 ♂, Xuelinzi, Nansi, Yaoba, Mt Helan, Alxa Left Banner, Inner Mongolia, 31.vii.2010, 2204 m (gen. slide no. 92/14, O Bidzilya), coll. Hongxia Liu and Zhiwei Zhang; 1 ♀, Xiangchizi Station, Mogou, Mt. Helan, Alxa Left Banner, Inner Mongolia, 1845 m, 11.viii.2011 (gen. slide no. 43/14, O Bidzilya), coll. Lixia Li and Yinghui Mu; 1 ♀, Ganjiahu Haloxylon Forest National Nature Reserve, Tacheng, Xinjiang Uigur Autonomous Region, 14.ix.1984 (gen. slide no. 276/08, O Bidzilya).

**Distribution.** China (Inner Mongolia, Gansu, Xinjiang), Mongolia.

**Remarks.** This species is recorded from China for the first time.

**13. *Scrobipalpacandicans* (Povolný, 1996)**

**Distribution**. China (Xinjiang), Kyrgyzstan, SE Kazakhstan.

**14. *Scrobipalpafusca* Bidzilya & Li, 2010**

**Distribution.** China (Inner Mongolia, Xinjiang), SE Kazakhstan, Turkmenistan, Uzbekistan.

**15. *Scrobipalpafrugifera* Povolný, 1969**

**Material examined**. 1 ♀, East Ujimqin Banner, Inner Mongolia, 8.viii.1997, 920 m, coll. Houhun Li (gen. slide no. L14033).

**Distribution.** China (Inner Mongolia), Russia (Kalmykia, southern Siberia from Altai to Khabarovskiy kray) (Anikin and Piskunov 2003; Ponomarenko 2008; Bidzilya 2009), Mongolia.

**Remarks.** This species is recorded from China for the first time.

**16. *Scrobipalplapsammophila* sp. n.**

**Distribution.** China (Ningxia).

**17. *Scrobipalpamongolica* Povolný, 1969**

**Distribution**. China (Inner Mongolia, Qinghai, Xinjiang), Mongolia.

**18. *Scrobipalpamongoloides* Povolný, 1969**

**Distribution**. China (Gansu, Hebei, Henan, Inner Mongolia, Ningxia, Qinghai, Xinjiang), Kazakhstan, Kyrgyzstan, North Pakistan, Uzbekistan, Mongolia.

**19. *Scrobipalpaaganophthalma* (Meyrick, 1931)**

**Distribution**. China (Tibet).

**20. *Scrobipalpanigrigrisea* Bidzilya & Li, 2010**

**Distribution.** China (Tibet).

**21. *Scrobipalpasolitaria* Povolný, 1969**

**Distribution**. China (Xinjiang). Ukraine, Russia (S Ural), Mongolia.

**22. *Scrobipalpamaniaca* Povolný, 1969**

**Distribution.** China (Xinjiang), Afghanistan, Mongolia, Russia (Lower Volga region, Zabaikalskiy krai), Turkmenistan, Uzbekistan (Falkovitsh and Bidzilya 2009).

**23. *Scrobipalpaintima* (Povolný, 2001)**

**Distribution**. China (Hebei), Russia (Zabaikalskiy krai).

**24. *Scrobipalpasubnitens* Povolný, 1969**

*Scrobipalpasubnitens* Povolný, 1969: 18. Type Locality: Dalanzadgad, S. Gobi Aimak, Mongolia.

*Scrobipalpagorodkovi* Bidzilya, 2012, syn. n.

**Material examined**. 1 ♂, Liuyangpu, Yanchi County (37°47’ N, 107°23’ E), Ningxia Hui Autonomous Region, 16.vi.2014, 1320 m, coll. Houhun Li, Wei Gaun and Meiqing Yang (gen. slide no. L13073).

**Distribution.** China (Ningxia), Mongolia, Tadzhikistan.

**Remarks.***Scrobipalpagorodkovi* was described from two males and one female from the mountains of Tadzhikistan (Bidzilya 2012). We compared the male genitalia of the paratype of *S.subnitens* in unrolled position with those of *S.gorodkovi* and came to the conclusion that they are conspecific. The female genitalia of both species are also identical, so that the following synonymy is established: *S.gorodkovi* Bidzilya, 2012 syn. n. of *S.subnitens* Povolný, 1969. This species is recorded from China for the first time.

**25. *Scrobipalpapeteri* Bidzilya, 2009**

**Material examined**. 1 ♀, Xiyaocun, Ningwu County, Shanxi Province, 1475 m, 21.vii.2011, coll. Shulian Hao and Jiayu Liu (gen. slide no. 79/14, O. Bidzilya).

**Distribution.** China (Shanxi), Russia (Tuva).

**Remarks.** This species is recorded from China for the first time.

**26. *Scrobipalpaindignella* (Staudinger, 1879)**

**Distribution**. China (Xinjiang), Afghanistan, Turkmenistan, Syria, Azerbaijan, Russia (SE of the European part), south Ukraine.

**27. *Scrobipalpaningxica* sp. n.**

**Distribution.** China (Ningxia).

**28. *Scrobipalpakurokoi* Povolný, 1977**

**Material examined**. 1 ♂, Qiuqianjia, Mt. Liupan, Ningxia Hui Autonomous Region, 1.vii.2008, 1700 m (gen. slide no. 103/15, O Bidzilya), coll. Shulian Hao and Zhiwei Zhang; 1 ♂, Heshangmaozi, Benxi County, Liaoning Province, 23.vi.2010 (gen. slide no. 123/14, O Bidzilya), coll. Jiayu Liu and Yanpeng Cai; 1 ♂, Baotainman, Neixiang County, Henan Province, 1200 m, 30.v.2006, coll. (gen. slide no. 97/15, O Bidzilya).

**Distribution.** China (Henan, Liaoning, Ningxia), Japan, Russia (Sakhalin Island) (Ponomarenko and Dubinina 2011).

**Remarks**. This species is recorded from China for the first time.

**29. *Scrobipalpagobica* Povolný, 1969**

**Distribution**. China (Xinjiang), Mongolia.

**30. *Scrobipalpatripunctella* sp. n.**

**Distribution.** China (Hebei, Ningxia, Shanxi).

**31. *Scrobipalpaavetjanae* Emelyanov & Piskunov, 1982**

**Material examined**. 5 ♂, 23 ♀, Ganshuwan, Gulaben, Mt. Helan, Alxa Left Banner, Inner Mongolia, 9.viii.2010, 2250 m, coll. Hongxia Liu and Zhiwei Zhang (gen. slide nos. L13044♂; L13045♀); 1 ♂, Xuelinzi, Nansi, Yaoba, Mt. Helan, Alxa Left Banner, Inner Mongolia, 31.vii.2010, 2204 m, coll. Hongxia Liu and Zhiwei Zhang; 1 ♂, Xuelinzi, Nansi, Yaoba, Mt. Helan, Alxa Left Banner, Inner Mongolia, 2204 m, 31.vii.2010, coll. Hongxia Liu and Zhiwei Zhang; 1 ♂, Beisi, Mogou, Mt. Helan, Alxa Left Banner, Inner Mongolia, 2027 m, 6.viii.2010 (gen. slide no. L14013), coll. Hongxia Liu and Zhiwei Zhang; 1 ♀, Xining City, Qinghai Province, 28.vii.1994 (gen. slide no. L06100); 1 ♀, Minqing, Gansu Province, 1343 m, 26.vii.2006, coll. Xinpu Wang and Xiangfeng Shi (gen. slide no. L07043).

**Distribution.** China (Gansu, Inner Mongolia, Qinghai), Armenia, Mongolia.

**Remarks.** This species is recorded from China for the first time.

**32. *Scrobipalpaparki* Povolný, 1993**

**Distribution**. China (Gansu, Hebei, Ningxia, Qinghai, Shaanxi, Xinjinag), South Korea.

**33. *Scrobipalpacoctans* Povolný, 1969**

**Distribution**. China (Shaanxi; Mongolia).

**34. *Scrobipalpaseptentrionalis* sp. n.**

**Distribution.** China (Heilongjiang, Ningxia).

**35. *Scrobipalpapauperella* (Heinemann, 1870)**

**Distribution**. China (Gansu, Ningxia, Inner Mongolia), Europe, Afghanistan, Russia (Zabaikalskiy krai).

**36. *Scrobipalpaliui* sp. n.**

**Distribution.** China (Shanxi).

**37. *Scrobipalpasimilis* Povolný, 1973**

**Distribution**. China (Gansu, Xinjiang), Mongolia, Russia (Zabaikalskiy krai), SE Kazakhstan.

**38. *Scrobipalpaochrostigma* Bidzilya & Li, 2010**

**Distribution.** China (Gansu, Qinghai).

**39. *Scrobipalpabryophiloides* Povolný, 1966**

**Distribution**. China (Xinjiang, Shaanxi, Ningxia, Inner Mongolia), south Ukraine, south of European part of Russia, Turkey, Uzbekistan, Iran, Mongolia, Kazakhstan, Uzbekistan, Turkmenistan.

**40. *Scrobipalpaartemisiella* (Treitschke, 1833)**

**Distribution**. Palaearctic Region to China (Xinjiang, Ningxia) and Mongolia eastwards.

**41. *Scrobipalpalatiuncella* Bidzilya & Li, 2010**

**Distribution**. China (Ningxia).

**42. *Scrobipalpaselectella* (Caradja, 1920)**

**Distribution**. South of Palaearctic Region from Tunisia to China (Xinjiang, Ningxia, Inner Mongolia, Tianjin).

**43. *Scrobipalpadistincta* Bidzilya & Li, 2010**

**Distribution**. China (Gansu, Henan, Ningxia).

**44. *Scrobipalpagozmanyi* Povolný, 1969**

**Distribution**. China (Xinjiang, Qinghai, Ningxia, Gansu, Sichuan), Mongolia.

**45. *Scrobipalpadivergens* (Povolný, 2002)**

**Distribution**. China (Xinjiang).

**46. *Scrobipalpapulchra* Povolný, 1967**

**Distribution**. Latvia (Huemer and Karsholt 2010: 167), South of Palaearctic Region from southern Ukraine and Turkey to Mongolia and west China (Xinjiang).

**47. *Scrobipalpahonei* Bidzilya & Li, 2010**

**Distribution.** China (Yunnan).

**48. *Scrobipalpastrictella* Bidzilya & Li, 2010**

**Distribution**. China (Hebei).

**49. *Scrobipalpaheretica* Povolný, 1973**

**Distribution.** China (Xinjiang); Spain, Russia (Volga and Ural regions), Turkey, Iran, Kazakhstan, Kyrgyzstan.

**50. *Scrobipalpamagnificella* Povolný, 1967**

**Distribution**. China (Xinjiang), south Ukraine, Russia (southern Ural mountains), Syria, north Iran, Uzbekistan, Mongolia.

**51. *Scrobipalpaflavimaculata* Bidzilya & Li, 2010**

**Distribution.** China (Qinghai).

**52. *Scrobipalpaflavidinigra* Bidzilya & Li, 2010**

**Distribution**. China (Inner Mongolia, Ningxia).

**53. *Scrobipalpanitentella* (Fuchs, 1902)**

**Distribution.** China (Qinghai, Xinjiang), Europe, Turkey, Kazakhstan, Afghanistan, Russia (European part, Novosibirsk region, Zabaikalskiy krai), Mongolia.

**54. *Scrobipalpaahasver* Povolný, 1969**

**Material examined.** 2 ♀, Weihugou, Jimsar County, Xinjiang Uigur Autonomous Region, 10.viii.2007, 1734 m (gen. slide nos. 87/14, O Bidzilya; L14038).

**Distribution.** China (Xinjiang), Mongolia.

**Remarks.** The genitalia of two females match well the figures by Povolný (2002: pl. 60, fig. 552), although the adults are lighter, greyish-black rather than dark brown and have a reduced subapical fascia on the forewing.

This species is recorded from China for the first time.

**55. *Scrobipalpacaryocoloides* Povolný, 1977**

**Distribution.** Japan, China (Ningxia, Shaanxi), Korea.

**56. *Scrobipalpachitensis* Povolný, 2001**

**Distribution**. China (Shaanxi, Ningxia), Russia (Zabaikalskiy krai).

**56. *Scrobipalpabidzilyai* (Povolný, 2001)**

**Distribution**. China (Hebei), Russia (Burjatia, Zabaikalskiy krai).

**57. *Scrobipalpatriangulella* sp. n.**

**Distribution.** China (Gansu, Ningxia, Shaanxi).

**58. *Scrobipalpapunctulata* sp. n.**

**Distribution.** China (Henan, Shanxi).

**59. *Scrobipalpachinensis* Povolný, 1969**

**Distribution**. China (Yunnan).

**60. *Scrobipalpasinica* Bidzilya & Li, 2010**

**Distribution**. China (Inner Mongolia; Mongolia).

**61. *Scrobipalpaflavinerva* Bidzilya & Li, 2010**

(Fig. 30)

**Distribution.** China (Ningxia, Inner Mongolia), Mongolia, Russia (Buryatia) (Bidzilya and Nupponen 2018).

**62. *Scrobipalpazouhari* Povolny, 1984**

**Distribution**. China (Beijing).

**63. *Scrobipalparebeli* (Preissecker, 1914)**

**Distribution**. China (Shaanxi), Europe (Italy, Austria, Ukraine), Russia (Tuva), Japan.

**64. *Scrobipalpanigrosparsea* Povolný, 1969**

**Distribution**. China (Inner Mongolia, Xinjiang), Mongolia.

**65. *Scrobipalpaerichi* Povolný, 1964**

**Distribution**. China (Xinjiang, Inner Mongolia), Europe, Iran, Turkey, Middle Asia, Mongolia.

**66. *Scrobipalpaerichiodes* Bidzilya & Li, 2010**

**Distribution**. China (Gansu, Hebei, Heilongjiang, Inner Mongolia, Ningxia, Shaanxi, Xinjiang).

**67. *Scrobipalpasattleri* Lvovsky & Piskunov, 1989**

**Distribution**. China (Inner Mongolia), Turkmenistan, Mongolia.

**68. *Scrobipalpanigripuncta* Bidzilya & Li, 2010**

**Distribution**. China (Henan).

**69. *Scrobipalplazhongweina* sp. n.**

**Distribution.** China (Ningxia).

**70 . *Scrobipalpahalimioniella* Huemer & Karsholt, 2010**

**Material examined.** 1 ♂, Burqin County, Xinjiang Uigur Autonomous Region, 21.vii.2007, 504 m, coll. Xinpu Wang (gen. slide no. 123/13, O Bidzilya).

**Distribution.** China (Xinjiang), south France, sSouth Ukraine (Bidzilya et al. 2011).

**Remarks.** The specimen matches *S.halimionella* well, both externally and in the genitalia, as figured in the original description (see Huemer and Karsholt 2010, fig. 110). This is a rather surprising discovery of this Mediterranean species, so far away from its previously known range.

This species is recorded from China for the first time.

**71. *Scrobipalpaergasima* (Meyrick, 1916)**

**Material examined**. 1 ♂, Erdaohu, Habahu, Yanchi County, Ningxia Hui Autonomous Region, 1397 m, 20.vii.2013, coll. Houhun Li (gen. slide no. 120/15, O Bidzilya).

**Distribution.** South of the Palaearctic Region eastwards to China (Ningxia) and Japan, arid areas of Africa and south-eastern Asia.

**Remarks.** This species is recorded from China for the first time.

## Supplementary Material

XML Treatment for Scrobipalpa triangulella

XML Treatment for Scrobipalpa punctulata

XML Treatment for Scrobipalpa septentrionalis

XML Treatment for Scrobipalpa zhongweina

XML Treatment for Scrobipalpa tripunctella

XML Treatment for Scrobipalpa ningxica

XML Treatment for Scrobipalpa psammophila

XML Treatment for Scrobipalpa flavinerva

XML Treatment for Scrobipalpa zhengi

XML Treatment for Scrobipalpa liui

## References

[B1] Anikin VV Piskunov VI (2003) Species of gelechiid moths (Lepidoptera, Gelechiidae), new to the European fauna. Povolzhskii ekologicheskii zhurnal 1: 70–71. [in Russian, with English abstract]

[B2] Bidzilya AV Budashkin YuI Zhakov AV Kostjuk IYu (2011) New and interesting records of Microlepidoptera (Lepidoptera) from Ukraine. Eversmannia 25/26: 64–74. [in Russian, with English abstract]

[B3] Bidzilya O (2009) On the distribution of gelechiid moths (Lepidoptera, Gelechiidae) in Siberia. Proceedings of Zoological Museum of Kiev Taras Shevchenko National University 5: 3–13.

[B4] Bidzilya O (2012) Two new species of the tribe Gnorimoschemini (Lepidoptera, Gelechiidae) from Palaearctic Asia. Entomofauna 33(11): 157–164.

[B5] Bidzilya OV Li HH (2010) The genus *Scrobipalpa* Janse (Lepidoptera, Gelechiidae) in China, with description of 13 new species. Zootaxa 2513: 1–26. 10.11646/zootaxa.2513.1.1

[B6] Bidzilya OV Li HH (2016) A review of the genus *Kiwaia* Philpott, 1930 (Lepidoptera, Gelechiidae) in the Palaearctic Region. Zootaxa 4098(3): 471–497. 10.11646/zootaxa.4098.3.3 27394596

[B7] Bidzilya O Nupponen K (2018) New species and new records of gelechiid moths (Lepidoptera, Gelechiidae) from southern Siberia. Zootaxa 4444(4): 381–408. 10.11646/zootaxa.4444.4.2 30313913

[B8] Falkovitsh M Bidzilya O (2006) The Turanian gelechiid moths of the tribe Gnorimoschemini (Lepidoptera, Gelechiidae) living on plants of the family Chenopodiaceae, with descriptions of new species. Proceedings of Zoological Museum of Kiev Taras Shevchenko National University 4: 62–104 [in Russian, with English abstract]

[B9] Falkovitsh MI Bidzilya OV (2009) A list of gelechiid moths (Lepidoptera, Gelechiidae) of the southern Kyzylkum. Proceedings of Zoological Museum Kiev Taras Shevchenko National University 5: 65–98.

[B10] Huemer P (1988) A taxonomic revision of *Caryocolum* (Lepidoptera, Gelechiidae). Bulletin of the British Museum (Natural History), Entomology 57: 439–571.

[B11] Huemer P Karsholt O (2010) Gelechiidae II (Gelechiinae: Gnorimoschemini). In: Huemer P, Karsholt O, Nuss M (Eds) Microlepidoptera of Europe 6. Apollo Books, Stenstrup, 586 pp 10.1002/mmnd.4800460215

[B12] Lee S Hodges RW Brown RL (2009) Checklist of Gelechiidae (Lepidoptera) in America north of Mexico. Zootaxa 2231: 1–39.

[B13] Li HH (2002) The Gelechiidae of China (I) (Lepidoptera, Gelechioidea). Nankai University Press, Tianjin, 538 pp. [in Chinese, with English summary]

[B14] Li HH Bidzilya O (2008) A review of the genus *Ephysteris* Meyrick, 1908 from China, with descriptions of two new species (Lepidoptera: Gelechiidae). Zootaxa 1733: 45–56. 10.3897/nl.41.23395

[B15] Li HH Bidzilya O (2017) Review of the genus *Gnorimoschema* Busck, 1900 (Lepidoptera, Gelechiidae) in China. Zootaxa 4365(2): 173–195. 10.11646/zootaxa.4365.2.4 29686216

[B16] Nazari V Landry J-F (2012) Gnorimoschemini fauna of Alberta (Lepidoptera: Gelechiidae). Report prepared for the Alberta Lepidopterists’ Guild. AB, Edmonton, 117 pp http://www.albertalepguild.ca/projects/faunalinventories/

[B17] Park KT Ponomarenko MG (2006) New faunistic data for the family Gelechiidae in the Korean peninsula and NE China (Lepidoptera: Gelechiidae). SHILAP Revista de Lepidopterologia 34(135): 275–288.

[B18] Pitkin LM (1986) A technique for the preparation of complex male genitalia in Microlepidoptera. Entomologist’s Gazette 37: 173–179.

[B19] Ponomarenko MG (2008) Gelechiidae. In: Sinev SYu (Ed.) Catalogue of the Lepidoptera of Russia.KMK Scientific Press, St. Petersburg/Moscow, 87–106. [in Russian, with English abstract]

[B20] Ponomarenko MG Dubinina VA (2011) New records of the Gelechioid moths (Lepidoptera: Gelechioidea) from Sakhalin island. Far Eastern Entomologist 223: 1–7.

[B21] Povolný D (1977) Notes on Gnorimoschemini of Australia and New Zealand. Acta entomologica Musei nationalis Pragae 39: 403–443.

[B22] Povolný D (1990) Zur heutigen Kenntnis von Nahrungspflanzen der Tribus Gnorimoschemini. Acta Universitatis Agriculturae (Brno) 38: 194–204.

[B23] Povolný D (1991) Zur Bionomie und Ökonomischen Bedeutung der Tribus Gnorimoschemini (Lepidoptera, Gelechiidae). Acta Universitatis Agriculturae (Brno) 39: 269–285.

[B24] Povolný D (2002) Iconographia tribus Gnorimoschemini (Lepidoptera, Gelechiidae) Regionis Palaearcticae. F Slamka publisher, Bratislava, 110 pp. [16 colour pls, 87 pls]

